# Novel immune-nutritional prognostic ratio predicts long-term survival in stage I–III colorectal cancer

**DOI:** 10.3389/fonc.2025.1694587

**Published:** 2025-12-18

**Authors:** Kuan Wang, Boxiang Zhang, Kejin Li, Ziyi Zhang, Xiangyue Zeng, Jun-Min Guan, Richard Aldridge, Elizabeth Whitmore, Yipeng Pan, Lucy Yue Lau, Zeliang Zhao, Yi Chen

**Affiliations:** 1 Department of Gastrointestinal Surgery, The Affiliated Cancer Hospital of Xinjiang Medical University, Urumqi, China; 2 Cancer Research Institute, The Affiliated Cancer Hospital of Xinjiang Medical University, Urumqi, Xinjiang, China; 3 Xinjiang Key Laboratory of Translational Biomedical Engineering, Urumqi, Xinjiang, China; 4 Department of Oncology, Nanfang Hospital, Southern Medical University, Guangzhou, Guangdong, China; 5 Department of Gastrointestinal Oncology Surgery, Gastroenterology Center, People’s Hospital of Bortala Mongolian Autonomous Prefecture, Bole, China; 6 Department of Medicine, University of Minnesota, Minneapolis, MN, United States; 7 Department of Gastroenterology, Sir Run Run Shaw Hospital, Zhejiang University School of Medicine, Hangzhou, Zhejiang, China; 8 Department of Public Health, Harvard Medical School, Boston, MA, United States

**Keywords:** colorectal cancer, overall survival, prognosis, nomogram, lymphocyte-to-monocyte ratio

## Abstract

**Background:**

Colorectal cancer (CRC) is a common and highly lethal malignancy worldwide. Even after curative resection, patients with stage I–III disease remain at substantial risk of recurrence and mortality. The Prognostic Immune and Nutritional Index (PINI) and lymphocyte-to-monocyte ratio (LMR) have been validated as prognostic markers in cancer, yet their individual predictive performance remains limited. We developed a novel Immune-Nutritional Prognostic Ratio (INPR) integrating PINI and LMR to provide a more comprehensive assessment of immune, nutritional, and inflammatory status. This study further evaluated its value in predicting 1-, 3-, and 5-year survival in stage I–III CRC.

**Methods:**

We retrospectively analyzed data from 556 colorectal cancer patients at two hospitals, with one serving as the validation cohort. Receiver operating characteristic (ROC) curves were used to determine optimal cutoff values for PINI and LMR, and the area under the curve (AUC) was applied to assess predictive performance. KKaplan–Meier analysis showed that lower PINI and LMR were associated with shorter overall survival (OS). The INPR, integrating both markers, demonstrated superior accuracy. Variables linked to OS were selected using the Boruta algorithm and multivariable Cox regression, and a nomogram model was developed and validated internally and externally.

**Results:**

The Youden index identified optimal cutoff values of 3.50 for PINI and 2.65 for LMR, with low levels independently predicting shorter OS. The INPR, integrating both, stratified patients into low-, intermediate-, and high-risk groups, with 5-year OS rates of 93.30%, 59.35%, and 28.57% in the training cohort (p<0.001). INPR outperformed either marker alone, showing higher AUC. A nomogram incorporating variables selected by the Boruta algorithm and multivariable Cox regression demonstrated stable and superior prognostic performance in both internal and external validation.

**Conclusion:**

Our findings demonstrate that INPR is a simple, accessible, and effective prognostic tool for postoperative risk stratification in stage I–III CRC patients, providing valuable guidance for optimizing individualized treatment strategies.

## Introduction

According to the latest global cancer statistics, colorectal cancer (CRC) ranks as the third most common malignancy, accounting for approximately 9.6% of all newly diagnosed cancers. It is also the second leading cause of cancer-related death, responsible for about 9.3% of cancer mortality worldwide (1). Despite advances in surgical techniques, perioperative management, and adjuvant therapies in recent years, the long-term prognosis of patients with stage I–III CRC remains unsatisfactory (2, 3). Even after curative resection, 20%–40% of patients experience recurrence or metastasis within five years (4–6). Therefore, accurately identifying high-risk patients in the early postoperative period and optimizing follow-up frequency and individualized treatment strategies are critical challenges in current clinical practice.

An increasing body of evidence indicates that the host’s systemic inflammatory response and nutritional status play pivotal roles in tumor initiation, progression, and therapeutic response (6, 7). Inflammatory mediators can promote tumor cell proliferation, angiogenesis, and immune evasion, whereas malnutrition may impair immune surveillance and treatment tolerance (8–10). Composite indices that integrate inflammatory and nutritional parameters have attracted considerable attention because they are simple, low-cost, and highly reproducible, making them valuable tools for prognostic assessment in cancer.

Currently, multiple hematological inflammation- and nutrition-related biomarkers have been applied in CRC prognostic studies, including the neutrophil-to-lymphocyte ratio (NLR), platelet-to-lymphocyte ratio (PLR), and prognostic nutritional index (PNI) (11–14). These indices are easily obtainable, inexpensive, and reproducible, offering promising clinical applicability. However, their predictive accuracy can be influenced by acute stress, comorbidities, and treatment-related factors, limiting their generalizability. Moreover, most studies have focused on single markers, which fail to comprehensively reflect the multidimensional effects of inflammation, immunity, and nutrition.

The Prognostic Immune and Nutritional Index (PINI), calculated from serum albumin and monocyte counts, reflects the host’s nutritional reserves and immune status (15, 16). Low PINI levels have been associated with poor outcomes in gastrointestinal and other solid malignancies. Similarly, the lymphocyte-to-monocyte ratio (LMR) represents the balance between antitumor immune activity and tumor-promoting inflammatory responses (17, 18). A low LMR indicates reduced lymphocyte-mediated cytotoxicity and increased monocyte-driven tumor progression, which may lead to worse survival outcomes.

Although both PINI and LMR have demonstrated independent prognostic value in multiple studies, single indices remain limited in predictive power and may not capture the complex host–tumor interactions driving CRC progression. This limitation underscores the need for integrative approaches that combine multidimensional biological information to improve prognostic accuracy. Based on this rationale, we propose a novel composite index—the immune-nutritional prognostic ratio (INPR)—which integrates PINI and LMR into a single score. While PINI reflects the nutritional and immune baseline of the patient, LMR captures the dynamic inflammatory–immune balance influencing tumor progression. Combining these parameters may offer a more comprehensive characterization of a patient’s immune-nutritional status and inflammatory response, thereby improving risk assessment.

To date, no study has systematically evaluated the prognostic value of combining PINI and LMR in patients with stage I–III CRC after curative resection, nor has any study integrated the two into a single model to predict 1-, 3-, and 5-year survival. Our study integrates INPR with clinicopathological features to construct a nomogram model using the Boruta feature selection algorithm and multivariable Cox regression. Both internal and external validation were performed to assess its generalizability and clinical applicability.

This study aims to investigate the value of INPR in predicting overall survival (OS) after curative resection in patients with stage I–III CRC. We hypothesized that INPR would provide superior prognostic accuracy compared with single indices and that an INPR-based nomogram could serve as an effective tool for postoperative risk stratification, ultimately supporting more individualized follow-up and treatment strategies.

## Patients and methods

### Study population

**Figure 1:**
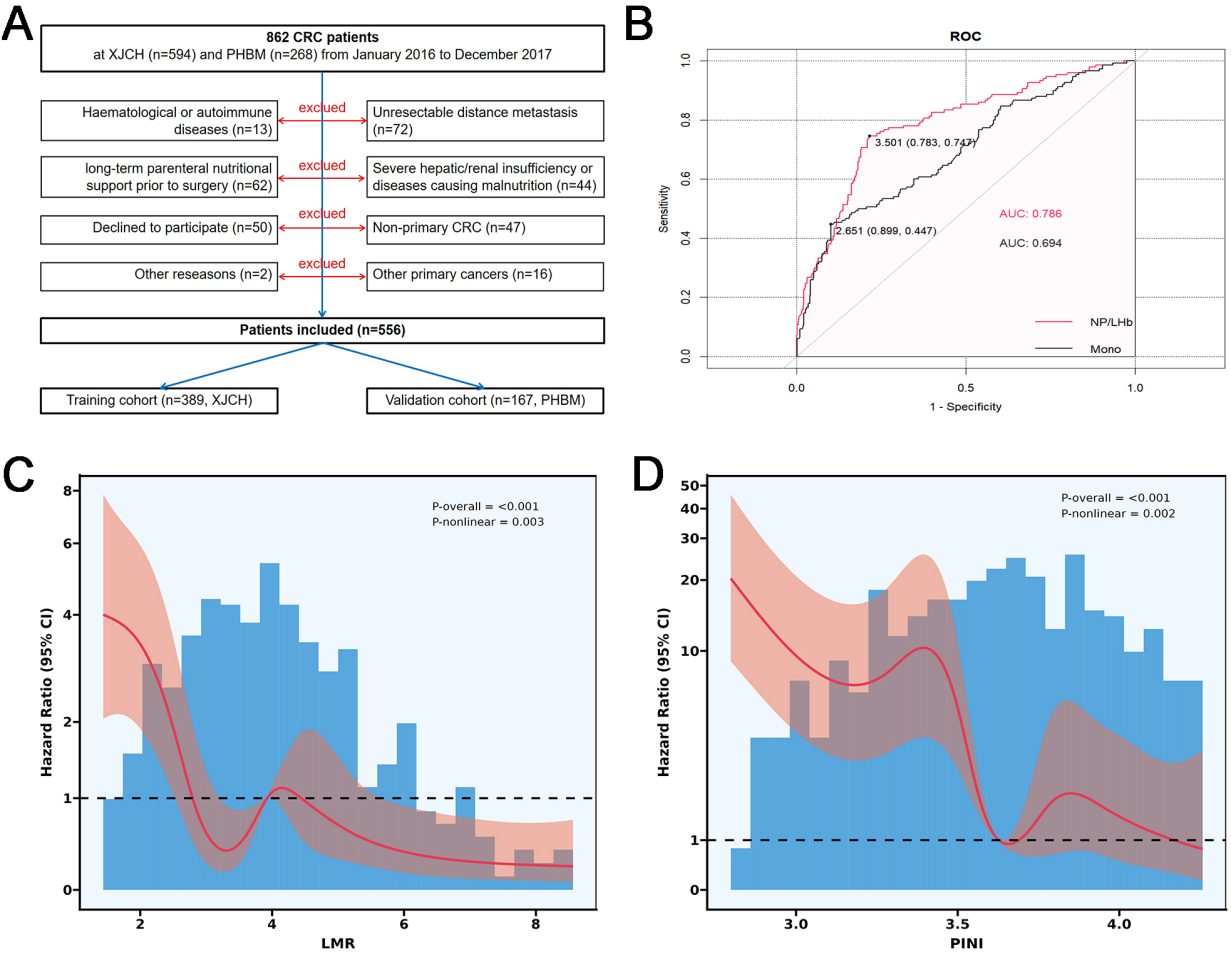
Study flowchart and determination of optimal cutoffs for LMR and PINI. **(A)** Flow diagram of patient enrollment, exclusion, and cohort allocation. **(B)** Receiver operating characteristic (ROC) curves of LMR and PINI for overall survival in the training cohort. **(C)** Restricted cubic spline (RCS) analysis showing the nonlinear association between LMR and overall survival. **(D)** RCS analysis showing the nonlinear association between PINI and overall survival.

From January 2016 to December 2017, a total of 862 patients with CRC were assessed for eligibility at two centers. After excluding 306 patients who did not meet the eligibility criteria, declined participation, or were removed for other reasons, 556 patients were finally included in the study. Among them, 389 patients from the Affiliated Cancer Hospital of Xinjiang Medical University (XJCH) comprised the training cohort, while 167 patients from the People’s Hospital of Bortala Mongolian Autonomous Prefecture (PHBM) in Xinjiang were assigned to the external validation cohort (**Figure 1A**). The inclusion criteria were as follows (1): primary CRC confirmed by postoperative histopathology; (2) radical surgical resection performed; (3) age > 18 years; (4) preoperative laboratory parameters available within 1 week before surgery; and (5) complete and reliable clinical data, with the ability to complete follow-up. The exclusion criteria were (1) having non-primary CRC; (2) with other primary cancers; (3) patients with unresectable distant metastases; (4) patients with hematological and autoimmune diseases; (5) patients with severe hepatic or renal insufficiency or diseases causing malnutrition; and (6) patients who received long-term parenteral nutritional support prior to surgery due to gastrointestinal dysfunction or malnutrition. Routine short-term fluid or glucose supplementation during the 24–48 hour preoperative fasting period was not considered parenteral nutrition.

The study was reviewed and approved by the Ethics Committee of the Affiliated Cancer Hospital of Xinjiang Medical University (Approval No. K-2024056) and the Ethics Committee of the People’s Hospital of Bortala Mongolian Autonomous Prefecture (Approval No. LLSH20241221). All procedures were conducted in accordance with the Declaration of Helsinki, and informed consent was obtained from all participants and/or their legal guardians. This study is a retrospective observational cohort analysis and does not constitute a clinical trial; therefore, trial registration was not required according to ICMJE and CONSORT guidelines.

### Data collection

Clinical and pathological data were retrospectively retrieved from the electronic medical record systems of the Affiliated Cancer Hospital of Xinjiang Medical University and the People’s Hospital of Bortala Mongolian Autonomous Prefecture in Xinjiang Uygur Autonomous Region, respectively. The dataset included: (1) demographic and baseline characteristics such as age, sex, height, weight, and history of smoking or alcohol consumption; (2) preoperative laboratory parameters obtained within 3–5 days before surgery, prior to the initiation of fasting or bowel preparation, including platelet, lymphocyte, neutrophil, and monocyte counts, hemoglobin, albumin, carcinoembryonic antigen (CEA), and carbohydrate antigen 19-9 (CA19-9) levels; (3) postoperative pathological findings, including tumor differentiation grade, vascular invasion, perineural invasion, and TNM classification; (4) follow-up information including survival status and survival time. Pathological staging was determined in accordance with the 8th edition (2018) of the American Joint Committee on Cancer (AJCC) Cancer Staging Manual.

Patients were followed up through outpatient visits and telephone contact every 3 months during the first 2 years after surgery and every 6 months thereafter. When telephone contact was unsuccessful, survival status was obtained from the national cancer registry. Follow-up lasted 60–80 months (median, 70 months). Patients lost to follow-up or alive without recurrence at the last follow-up were censored in the survival analysis.

Calculated from hematological indices: PINI = [albumin (g/dL) × 0.9] - [absolute monocyte count (/μL) × 0.0007]; LMR = lymphocytes count (10^9^/L)/monocytes count (10^9^/L).

### Statistical analysis

All statistical analyses were performed using SPSS software version 29.0 and R software version 4.4.1. Baseline clinical characteristics were summarized as follows: categorical variables were presented as counts and percentages and compared between groups using the chi-square test or Fisher’s exact test, as appropriate. Continuous variables were analyzed using the independent-samples t-test or one-way analysis of variance (ANOVA), while the Wilcoxon rank-sum test was applied for non-normally distributed or ordinal data. Differences in general clinical and pathological characteristics between the training and validation cohorts were also examined. ROC curve analysis was conducted in the training cohort to determine the optimal cutoff values for PINI and LMR, and the AUC was calculated to assess their predictive performance. These predefined thresholds were subsequently applied consistently in the validation cohort without recalibration. Kaplan–Meier survival curves were plotted for PINI and LMR, and the log-rank test was used to evaluate differences in OS. In addition, restricted cubic spline (RCS) models were applied to explore potential nonlinear associations between PINI, LMR, and mortality risk. A novel composite prognostic indicator, the INPR, was then established by integrating PINI and LMR, which represent complementary dimensions of systemic nutritional–inflammatory status and immune–inflammatory balance. Optimal cut-off values for PINI and LMR were determined using ROC curve analysis. Based on these thresholds, a novel composite prognostic indicator was established with the following scoring criteria: INPR = 0 (PINI < 3.50 and LMR < 2.65), INPR = 1 (PINI ≥ 3.50 or LMR ≥ 2.65), and INPR = 2 (PINI ≥ 3.50 and LMR ≥ 2.65). Patients were subsequently categorized into three biologically and clinically interpretable risk groups according to their combined PINI/LMR status, allowing for improved prognostic discrimination and clinical applicability. Univariate Cox regression analysis was first used to identify potential prognostic factors. To determine independent predictors of OS, we employed the Boruta algorithm in combination with multivariable Cox proportional hazards regression analysis. Based on the variables identified by these methods, a nomogram model was developed using the “rms” package in R to predict individual survival probabilities for CRC patients. The performance of the nomogram was assessed using ROC curves and calibration plots, and its predictive accuracy was validated in both the internal (training cohort) and external (validation cohort) datasets. The P value < 0.05 was considered statistically significant for all analyses. To assess potential multicollinearity between the composite indices integrated into the INPR model, variance inflation factor (VIF) and tolerance analyses were performed prior to multivariable modeling. The results demonstrated that all variables included in the construction of INPR exhibited low collinearity (all VIF values < 2.0), indicating no significant multicollinearity among PINI, LMR, or their constituent components. These findings support the statistical independence and methodological feasibility of integrating PINI and LMR into a composite prognostic indicator.

## Results

### Clinicopathological features of training and validation cohorts

A total of 556 patients diagnosed with CRC were included in this study, and their clinicopathological characteristics were collected and analyzed. As summarized in **Table 1**, the patients were divided into a training cohort (n = 389) and a validation cohort (n = 167). Baseline demographic and clinical characteristics were generally comparable between the two cohorts, with no statistically significant differences. Overall, 324 patients (58.3%) were male, and the median age was 62 years. Histories of smoking and alcohol consumption were present in 30.8% and 18.5% of patients, respectively. According to the TNM classification, 17.3% of patients were stage I, 44.8% were stage II, and 37.9% were stage III. Histologically, moderately differentiated adenocarcinoma was the predominant subtype (68.2%), followed by poorly differentiated (17.4%), undifferentiated (9.2%), and well-differentiated tumors (5.2%). Perineural invasion was observed in 94 patients (16.9%), and the same proportion of patients exhibited lymphovascular invasion. Regarding tumor markers, elevated CEA and CA19–9 levels were detected in 200 (36.0%) and 61 (11.0%) patients, respectively; these biomarkers are widely used to evaluate tumor burden and disease progression in CRC.

**Table 1:** Patient demographics and baseline characteristics.

Characteristic	Cohort			p-value^2^
	Overall N = 556^1^	Training Cohort N = 389^1^	Internal Test Cohort N = 167^1^	
Gender(n%)				0.286
female	232 (41.7%)	168 (43.2%)	64 (38.3%)	
male	324 (58.3%)	221 (56.8%)	103 (61.7%)	
Age(n%)				0.329
<45	54 (9.7%)	37 (9.5%)	17 (10.2%)	
45-59	186 (33.5%)	126 (32.4%)	60 (35.9%)	
60-74	244 (43.9%)	180 (46.3%)	64 (38.3%)	
≥75	72 (12.9%)	46 (11.8%)	26 (15.6%)	
BMI(n%)				0.348
<18.5	16 (2.9%)	12 (3.1%)	4 (2.4%)	
18.5-24	242 (43.5%)	178 (45.8%)	64 (38.3%)	
24-28	230 (41.4%)	152 (39.1%)	78 (46.7%)	
>28	68 (12.2%)	47 (12.1%)	21 (12.6%)	
Smoking(n%)				0.352
no	385 (69.2%)	274 (70.4%)	111 (66.5%)	
yes	171 (30.8%)	115 (29.6%)	56 (33.5%)	
Drink(n%)				0.484
no	453 (81.5%)	314 (80.7%)	139 (83.2%)	
yes	103 (18.5%)	75 (19.3%)	28 (16.8%)	
T stage(n%)				0.323
T1	27 (4.9%)	23 (5.9%)	4 (2.4%)	
T2	83 (14.9%)	57 (14.7%)	26 (15.6%)	
T3	398 (71.6%)	274 (70.4%)	124 (74.3%)	
T4	48 (8.6%)	35 (9.0%)	13 (7.8%)	
N stage(n%)				0.881
N0	345 (62.1%)	239 (61.4%)	106 (63.5%)	
N1	129 (23.2%)	91 (23.4%)	38 (22.8%)	
N2	82 (14.7%)	59 (15.2%)	23 (13.8%)	
Tumor stage(n%)				0.836
I	96 (17.3%)	68 (17.5%)	28 (16.8%)	
II	249 (44.8%)	171 (44.0%)	78 (46.7%)	
III	211 (37.9%)	150 (38.6%)	61 (36.5%)	
Differentiated degree(n%)				0.143
Moderately	379 (68.2%)	254 (65.3%)	125 (74.9%)	
Poorly	97 (17.4%)	75 (19.3%)	22 (13.2%)	
Undifferentiated	51 (9.2%)	37 (9.5%)	14 (8.4%)	
Well	29 (5.2%)	23 (5.9%)	6 (3.6%)	
Nerve invasion(n%)				0.425
no	462 (83.1%)	320 (82.3%)	142 (85.0%)	
yes	94 (16.9%)	69 (17.7%)	25 (15.0%)	
Intravascular tumor emboli(%)				0.124
no	462 (83.1%)	317 (81.5%)	145 (86.8%)	
yes	94 (16.9%)	72 (18.5%)	22 (13.2%)	
CEA				0.182
High	200 (36.0%)	133 (34.2%)	67 (40.1%)	
Normal	356 (64.0%)	256 (65.8%)	100 (59.9%)	
CA19-9				0.841
High	61 (11.0%)	42 (10.8%)	19 (11.4%)	
Normal	495 (89.0%)	347 (89.2%)	148 (88.6%)	

^1^n (%)

^2^Pearson's Chi-squared test; Fisher's exact test

### Association of PINI and LMR with clinicopathological characteristics in the training cohort

In the training cohort, PINI levels showed significant associations with several clinicopathological factors. Specifically, variations in PINI were significantly related to patient age (p = 0.003), alcohol consumption (p = 0.027), tumor T stage (p = 0.019), presence of perineural invasion (p = 0.002), and elevated CA19–9 levels (p = 0.004). Similarly, LMR levels were significantly correlated with T stage (p = 0.002) and overall TNM stage (p = 0.005). By contrast, neither PINI nor LMR demonstrated statistically significant associations with sex, body mass index (BMI), smoking status, N stage, tumor differentiation, vascular invasion, or CEA levels (**Table 2**).

**Table 2:** Association of preoperative CIPI and PAR with clinicopathological features in the training cohort.

Characteristic	PINI			p-value^2^	LMR			p-value^2^
	Overall N = 389^1^	<3.50 N = 136^1^	≥3.50 N = 253^1^		Overall N = 389^1^	<2.65 N = 71^1^	≥2.65 N = 318^1^	
Gender(n%)				0.710				0.217
female	168 (43.2%)	57 (41.9%)	111 (43.9%)		168 (43.2%)	26 (36.6%)	142 (44.7%)	
male	221 (56.8%)	79 (58.1%)	142 (56.1%)		221 (56.8%)	45 (63.4%)	176 (55.3%)	
Age(n%)				0.003				0.286
<45	37 (9.5%)	11 (8.1%)	26 (10.3%)		37 (9.5%)	9 (12.7%)	28 (8.8%)	
≥75	46 (11.8%)	25 (18.4%)	21 (8.3%)		46 (11.8%)	12 (16.9%)	34 (10.7%)	
45-59	126 (32.4%)	32 (23.5%)	94 (37.2%)		126 (32.4%)	19 (26.8%)	107 (33.6%)	
60-74	180 (46.3%)	68 (50.0%)	112 (44.3%)		180 (46.3%)	31 (43.7%)	149 (46.9%)	
BMI(n%)				0.286				0.403
<18.5	12 (3.1%)	7 (5.1%)	5 (2.0%)		12 (3.1%)	4 (5.6%)	8 (2.5%)	
>28	47 (12.1%)	16 (11.8%)	31 (12.3%)		47 (12.1%)	6 (8.5%)	41 (12.9%)	
18.5-24	178 (45.8%)	65 (47.8%)	113 (44.7%)		178 (45.8%)	34 (47.9%)	144 (45.3%)	
24-28	152 (39.1%)	48 (35.3%)	104 (41.1%)		152 (39.1%)	27 (38.0%)	125 (39.3%)	
Smoking(n%)				0.225				0.776
no	274 (70.4%)	101 (74.3%)	173 (68.4%)		274 (70.4%)	51 (71.8%)	223 (70.1%)	
yes	115 (29.6%)	35 (25.7%)	80 (31.6%)		115 (29.6%)	20 (28.2%)	95 (29.9%)	
Drink(n%)				0.027				0.574
no	314 (80.7%)	118 (86.8%)	196 (77.5%)		314 (80.7%)	59 (83.1%)	255 (80.2%)	
yes	75 (19.3%)	18 (13.2%)	57 (22.5%)		75 (19.3%)	12 (16.9%)	63 (19.8%)	
T stage(n%)				0.019				0.002
T1	23 (5.9%)	5 (3.7%)	18 (7.1%)		23 (5.9%)	0 (0.0%)	23 (7.2%)	
T2	57 (14.7%)	13 (9.6%)	44 (17.4%)		57 (14.7%)	5 (7.0%)	52 (16.4%)	
T3	274 (70.4%)	100 (73.5%)	174 (68.8%)		274 (70.4%)	55 (77.5%)	219 (68.9%)	
T4	35 (9.0%)	18 (13.2%)	17 (6.7%)		35 (9.0%)	11 (15.5%)	24 (7.5%)	
N stage(n%)				0.603				0.290
N0	239 (61.4%)	83 (61.0%)	156 (61.7%)		239 (61.4%)	38 (53.5%)	201 (63.2%)	
N1	91 (23.4%)	35 (25.7%)	56 (22.1%)		91 (23.4%)	21 (29.6%)	70 (22.0%)	
N2	59 (15.2%)	18 (13.2%)	41 (16.2%)		59 (15.2%)	12 (16.9%)	47 (14.8%)	
Tumor stage(n%)				0.072				0.005
I	68 (17.5%)	16 (11.8%)	52 (20.6%)		68 (17.5%)	3 (4.2%)	65 (20.4%)	
II	171 (44.0%)	67 (49.3%)	104 (41.1%)		171 (44.0%)	35 (49.3%)	136 (42.8%)	
III	150 (38.6%)	53 (39.0%)	97 (38.3%)		150 (38.6%)	33 (46.5%)	117 (36.8%)	
Differentiated degree(n%)				0.325				0.633
Moderately	254 (65.3%)	87 (64.0%)	167 (66.0%)		254 (65.3%)	43 (60.6%)	211 (66.4%)	
Poorly	75 (19.3%)	30 (22.1%)	45 (17.8%)		75 (19.3%)	17 (23.9%)	58 (18.2%)	
Undifferentiated	37 (9.5%)	9 (6.6%)	28 (11.1%)		37 (9.5%)	6 (8.5%)	31 (9.7%)	
Well	23 (5.9%)	10 (7.4%)	13 (5.1%)		23 (5.9%)	5 (7.0%)	18 (5.7%)	
Nerve invasion(n%)				0.002				0.242
no	320 (82.3%)	101 (74.3%)	219 (86.6%)		320 (82.3%)	55 (77.5%)	265 (83.3%)	
yes	69 (17.7%)	35 (25.7%)	34 (13.4%)		69 (17.7%)	16 (22.5%)	53 (16.7%)	
Intravascular tumor emboli(%)				0.295				0.530
no	317 (81.5%)	107 (78.7%)	210 (83.0%)		317 (81.5%)	56 (78.9%)	261 (82.1%)	
yes	72 (18.5%)	29 (21.3%)	43 (17.0%)		72 (18.5%)	15 (21.1%)	57 (17.9%)	
CEA				0.313				0.113
High	133 (34.2%)	51 (37.5%)	82 (32.4%)		133 (34.2%)	30 (42.3%)	103 (32.4%)	
Normal	256 (65.8%)	85 (62.5%)	171 (67.6%)		256 (65.8%)	41 (57.7%)	215 (67.6%)	
CA-199				0.004				0.778
High	42 (10.8%)	23 (16.9%)	19 (7.5%)		42 (10.8%)	7 (9.9%)	35 (11.0%)	
Normal	347 (89.2%)	113 (83.1%)	234 (92.5%)		347 (89.2%)	64 (90.1%)	283 (89.0%)	

^1^n (%)

^2^Pearson's Chi-squared test; Fisher's exact test

### Optimal cutoff determination and prognostic significance of PINI and LMR

In the training cohort, ROC curve analysis was first performed to assess the prognostic discriminatory ability of PINI and LMR and to determine their optimal cutoff points. The AUC of PINI was 0.786, with an optimal cutoff value of 3.50; for LMR, the AUC was 0.694, with an optimal cutoff of 2.65. Based on these thresholds, PINI values ≥3.50 and <3.50 were categorized into high (H-PINI) and low (L-PINI) groups, respectively; similarly, LMR values ≥2.65 and <2.65 were classified as high (H-LMR) and low (L-LMR) groups (**Figure 1B**). RCS analysis further demonstrated that decreasing levels of both PINI and LMR were associated with a progressively increasing risk of death, indicating a stable and significant prognostic relationship between these indices and OS (**Figures 1C, D**).

### Association of PINI and LMR with OS

**Figure 2:**
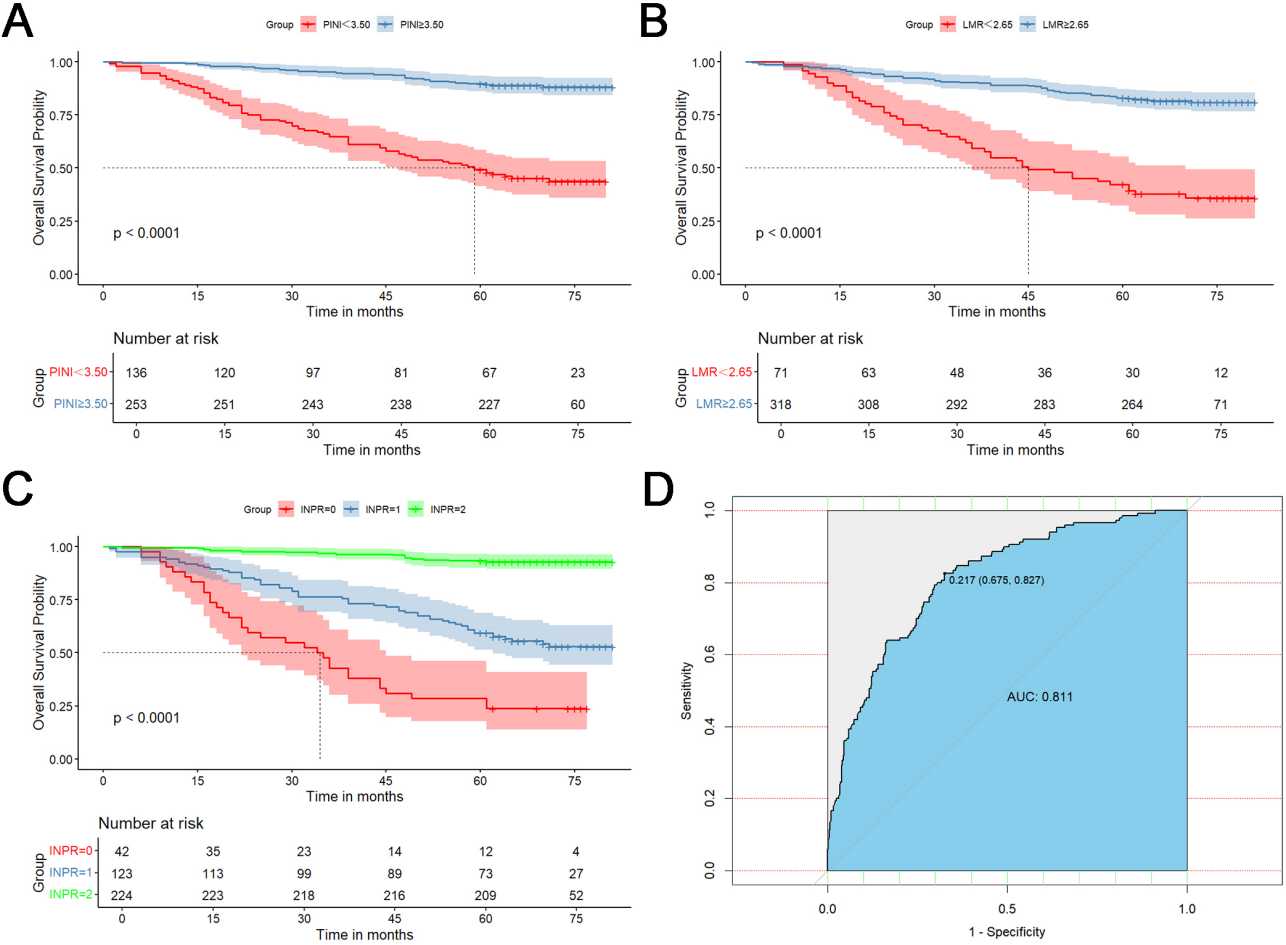
Kaplan–Meier survival analyses and predictive performance of INPR. **(A)** Overall survival (OS) stratified by PINI in the training cohort. **(B)** OS stratified by LMR in the training cohort. **(C)** OS stratified by INPR risk groups (INPR = 0, 1, 2). **(D)** Receiver operating characteristic (ROC) curve showing the predictive accuracy of INPR for OS.

In the training cohort, the median OS was 67 months. Kaplan–Meier survival analysis revealed that patients in the low PINI (L-PINI) group had significantly shorter OS than those in the high PINI (H-PINI) group (P < 0.001). Similarly, patients with low LMR (L-LMR) had significantly worse OS than those with high LMR (H-LMR) (P < 0.001). These findings indicate that low PINI or LMR levels are significantly associated with poorer OS in CRC patients (**Figures 2A, B**).

### INPR score stratification and association with survival outcomes

By integrating the prognostic information from PINI and LMR, we developed the INPR score (**Table 3**). In the training cohort of 389 patients, three distinct risk categories were defined: the high-risk group (INPR = 0), comprising patients with PINI < 3.50 and LMR < 2.65 (n = 42, 10.8%); the intermediate-risk group (INPR = 1), including those with PINI < 3.50 and LMR ≥ 2.65, or PINI ≥ 3.50 and LMR < 2.65 (n = 123, 31.62%); and the low-risk group (INPR = 2), consisting of patients with PINI ≥ 3.50 and LMR ≥ 2.65 (n = 224, 57.58%). Kaplan–Meier analysis revealed striking differences in 5-year OS across these groups, with survival rates of 28.57%, 59.35%, and 93.30%, respectively (P < 0.001; **Figure 2C**).

**Table 3:** Scoring criteria of the Immune-Nutritional Prognostic Ratio (INPR) based on PINI and LMR levels.

INPR	PINI	LMR	Number
0	<3.50	<2.65	224
1	PINI<3.50 or LMR<2.65		123
2	≥3.50	≥2.65	42

In addition, comparative analysis demonstrated that INPR provided superior prognostic discrimination compared with either PINI or LMR alone. INPR achieved an area under the ROC curve (AUC) of 0.811, outperforming both PINI (AUC = 0.786) and LMR (AUC = 0.694), indicating improved predictive accuracy (**Figure 2D**).

### Independent prognostic factors for OS in CRC patients

In the training cohort, univariate and multivariate Cox regression analyses were performed to evaluate clinicopathological variables associated with overall survival (OS) in CRC patients (**Table 4**). Univariate analysis revealed that BMI, N stage, TNM stage, tumor differentiation, nerve invasion, intravascular tumor emboli, CEA, PINI, LMR, and INPR were significantly associated with OS (P < 0.05). Multivariate analysis further identified tumor differentiation, CEA, PINI, LMR, and INPR as independent prognostic factors (P < 0.05). Specifically, patients with poorly differentiated tumors had a substantially higher risk of death compared with those with moderate differentiation (HR = 2.57, 95% CI: 1.55–4.27, P < 0.001). Elevated CEA levels were independently associated with shortened OS (HR = 2.53, 95% CI: 1.61–4.11, P < 0.001). A higher PINI (≥3.50) was associated with a significantly reduced mortality risk (HR = 0.57, 95% CI: 0.28–1.18, P = 0.030), while a higher LMR (≥2.65) independently predicted better OS (HR = 0.56, 95% CI: 0.33–0.96, P = 0.036). Regarding the novel scoring system, patients with INPR ≤1 exhibited a markedly higher risk of death compared with those with INPR = 2, with INPR demonstrating strong and consistent prognostic value in both univariate and multivariate analyses (HR = 0.17, 95% CI: 0.07–0.46, P < 0.001).

**Table 4:** Univariate and multivariate analysis of influencing factors (Cox regression).

Characteristic	Univariable					Multivariable				
	N	Event N	HR	95% CI	p-value	N	Event N	HR	95% CI	p-value
Gender(n%)										
female	168	43	—	—		168	43	—	—	
male	221	61	1.08	0.73, 1.60	0.687	221	61	1.08	0.66, 1.77	0.764
Age(n%)										
<45	37	10	—	—		37	10	—	—	
≥75	46	20	1.79	0.84, 3.82	0.133	46	20	1.90	0.84, 4.30	0.122
45-59	126	26	0.73	0.35, 1.52	0.406	126	26	0.91	0.42, 2.00	0.817
60-74	180	48	0.97	0.49, 1.91	0.920	180	48	1.28	0.60, 2.75	0.519
BMI(n%)										
<18.5	12	6	—	—		12	6	—	—	
>28	47	12	0.36	0.13, 0.95	0.039	47	12	0.76	0.25, 2.25	0.618
18.5-24	178	51	0.42	0.18, 0.99	0.047	178	51	0.76	0.28, 2.03	0.580
24-28	152	35	0.32	0.13, 0.76	0.010	152	35	0.68	0.26, 1.82	0.444
Smoking(n%)										
no	274	80	—	—		274	80	—	—	
yes	115	24	0.66	0.42, 1.04	0.072	115	24	0.41	0.21, 0.79	0.008
Drink(n%)										
no	314	88	—	—		314	88	—	—	
yes	75	16	0.70	0.41, 1.20	0.195	75	16	1.74	0.84, 3.60	0.134
T stage(n%)										
T1	23	2	—	—		23	2	—	—	
T2	57	6	1.22	0.25, 6.02	0.811	57	6	0.74	0.14, 3.96	0.725
T3	274	83	3.99	0.98, 16.21	0.053	274	83	0.79	0.09, 7.13	0.830
T4	35	13	5.00	1.13, 22.17	0.034	35	13	0.56	0.06, 5.47	0.615
N stage(n%)										
N0	239	48	—	—		239	48	—	—	
N1	91	32	1.90	1.21, 2.97	0.005	91	32	2.35	0.41, 13.41	0.338
N2	59	24	2.39	1.46, 3.90	<0.001	59	24	3.71	0.64, 21.60	0.144
Tumor stage(n%)										
I	68	6	—	—		68	6	—	—	
II	171	42	3.06	1.30, 7.19	0.010	171	42	1.66	0.27, 10.03	0.581
III	150	56	5.07	2.18, 11.76	<0.001	150	56			
Differentiated degree(n%)										
Moderately	254	55	—	—		254	55	—	—	
Poorly	75	35	2.77	1.81, 4.23	<0.001	75	35	2.57	1.55, 4.27	<0.001
Undifferentiated	37	8	1.07	0.51, 2.24	0.866	37	8	1.78	0.80, 3.96	0.158
Well	23	6	1.34	0.58, 3.11	0.500	23	6	1.85	0.73, 4.72	0.196
Nerve invasion(n%)										
no	320	72	—	—		320	72	—	—	
yes	69	32	2.56	1.69, 3.89	<0.001	69	32	1.66	0.95, 2.89	0.075
Intravascular tumor emboli(%)										
no	317	78	—	—		317	78	—	—	
yes	72	26	1.60	1.03, 2.49	0.038	72	26	1.12	0.64, 1.94	0.696
CEA										
Normal	256	56	—	—		256	56	—	—	
High	133	48	1.69	1.33, 2.47	<0.001	133	48	2.53	2.01, 5.11	<0.001
CA19-9										
Normal	347	87	—	—		347	87	—	—	
High	42	17	1.01	0.48, 2.17	0.980	42	17	1.53	0.69, 3.02	0.263
PINI										
<3.50	136	75	—	—		136	75	—	—	
≥3.50	253	29	0.15	0.10, 0.23	<0.001	253	29	0.57	0.28, 1.18	0.130
LMR										
<2.65	71	45	—	—		71	45	—	—	
≥2.65	318	59	0.21	0.14, 0.31	<0.001	318	59	0.56	0.33, 0.96	0.036
INPR										
≤1	165	88	—	—		165	88	—	—	
2	224	16	0.10	0.06, 0.17	<0.001	224	16	0.17	0.07, 0.46	<0.001

CI = Confidence Interval, HR = Hazard Ratio

**Figure 3:**
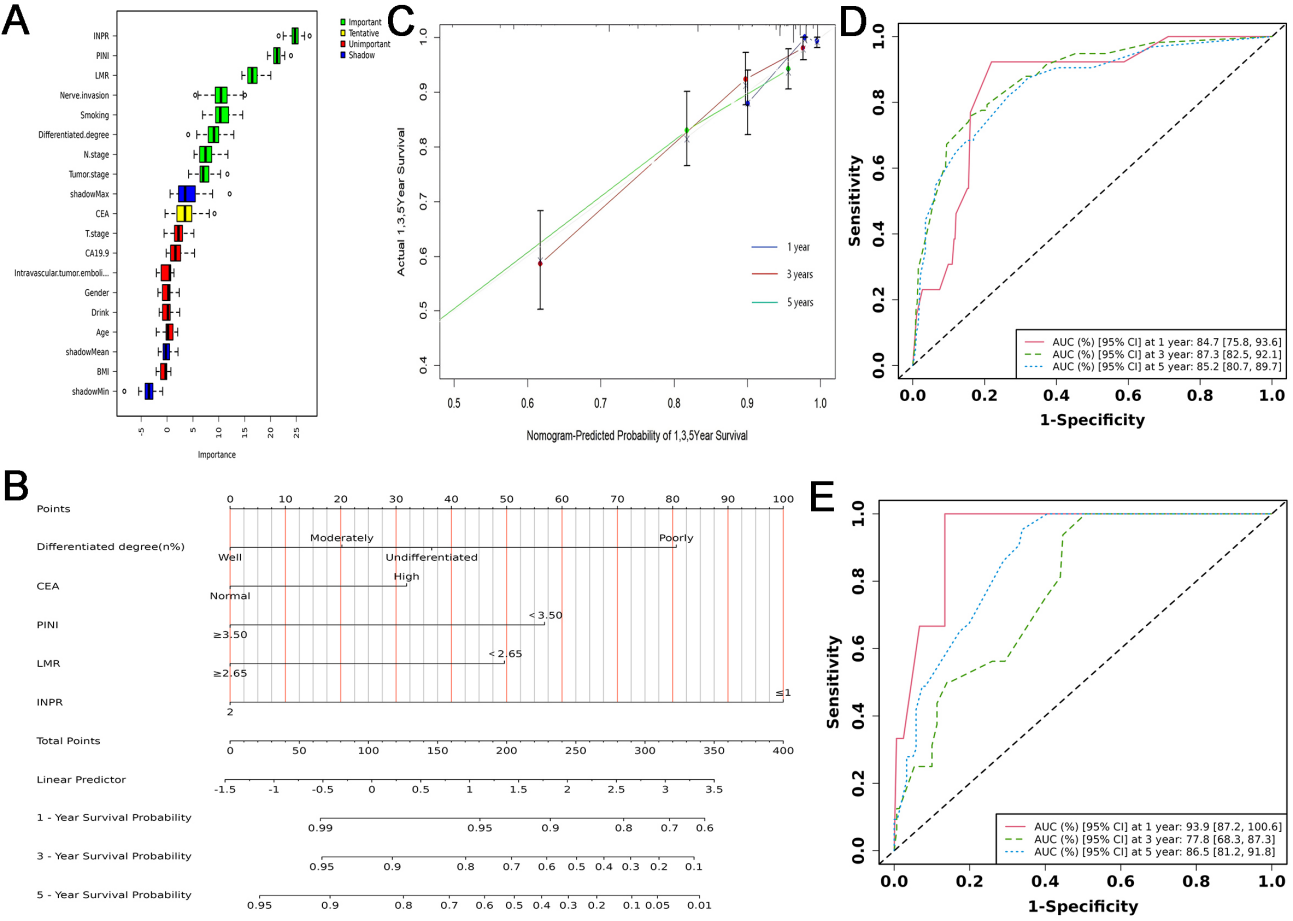
Construction and performance of the INPR-based nomogram. **(A)** Variable importance ranking for overall survival. **(B)** Nomogram integrating clinicopathological factors and INPR for predicting 1-, 3-, and 5-year overall survival. **(C)** Calibration curves of the nomogram at 1, 3, and 5 years. **(D)** Time-dependent ROC curves of the nomogram in the training cohort. **(E)** Time-dependent ROC curves of the nomogram in the validation cohort.

Collectively, these findings indicate that INPR offers superior prognostic stratification compared with traditional inflammatory and nutritional biomarkers. Based on this foundation, we further employed the Boruta algorithm to assess the relative importance of all candidate variables. The analysis identified eight variables as important contributors to OS, namely INPR, LMR, PINI, perineural invasion, tumor differentiation, tumor stage, N stage, and smoking status. In contrast, seven variables—including CA19-9, intravascular tumor emboli, T stage, drinking history, BMI, age, and sex—were not retained as important predictors. Notably, CEA fell into the “tentative” category as defined by the Boruta algorithm, suggesting uncertain relevance and the need for further evaluation (**Figure 3A**).

### Development and validation of a prognostic nomogram for OS prediction in CRC

Based on the multivariate Cox regression analysis and Boruta feature selection, five key variables—CEA, tumor differentiation, PINI, LMR, and INPR—were incorporated into the nomogram to estimate individualized prognostic risk in patients with CRC. Among these predictors, INPR and tumor differentiation contributed most prominently to survival prediction, with patients exhibiting INPR ≤ 1 or poor differentiation showing a markedly increased mortality risk. Moreover, lower PINI and LMR values and higher CEA levels were each significantly associated with worse prognosis (**Figure 3B**).

**Figure 4:**
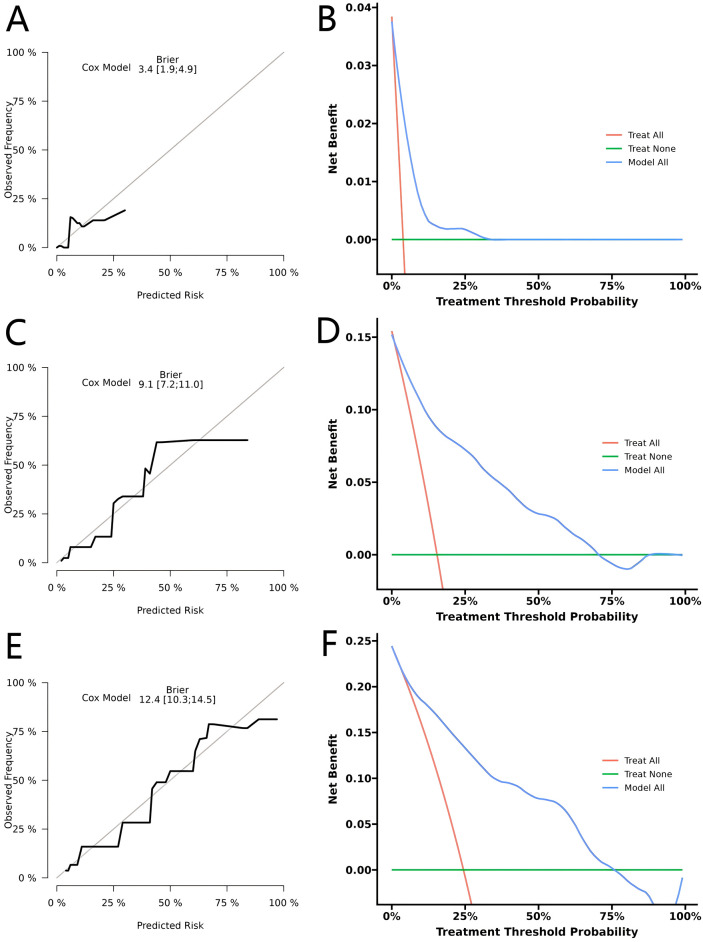
Calibration and decision curve analysis (DCA) for predicting 1-, 3-, and 5-year overall survival (OS) in the training cohort. **(A, C, E)** Calibration curves for 1-, 3-, and 5-year OS. **(B, D, F)** DCA curves for 1-, 3-, and 5-year OS.

For performance evaluation, we first performed internal validation. In the training cohort, time-dependent ROC analysis yielded AUCs of 0.847, 0.873, and 0.852 for predicting 1-, 3-, and 5-year OS, respectively, demonstrating strong discriminative performance (**Figure 3D**). Calibration curves showed excellent concordance between predicted and observed survival probabilities at all three time points (**Figure 3C**). In addition, the calibration plots and decision curve analyses for 1-, 3-, and 5-year OS (**Figures 4A–F**) consistently supported the nomogram’s high predictive accuracy and clinical utility.

**Figure 5:**
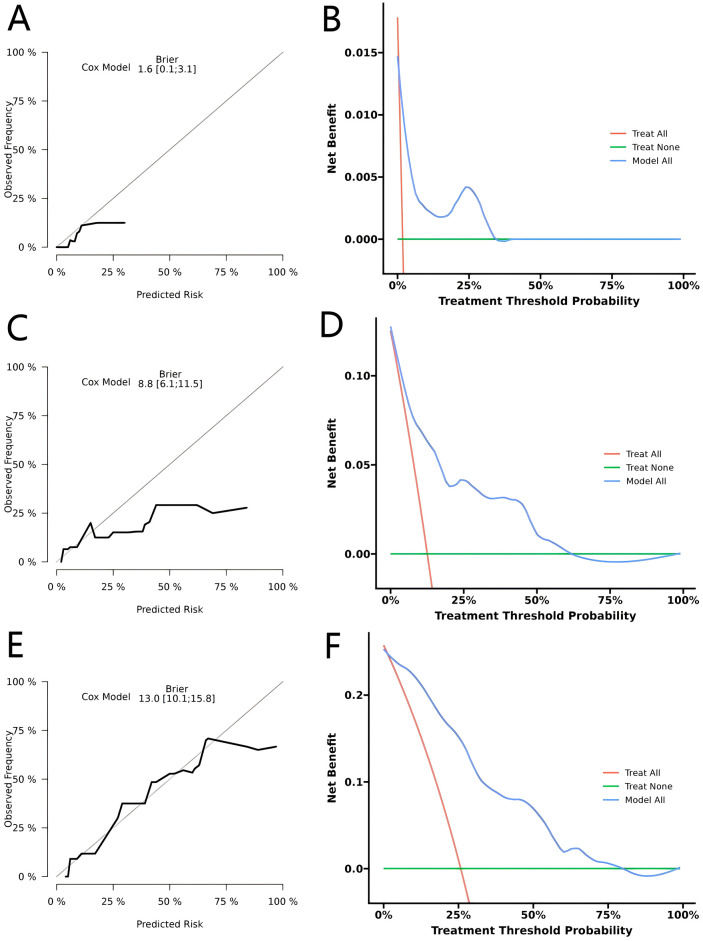
Calibration and decision curve analysis (DCA) for predicting 1-, 3-, and 5-year overall survival (OS) in the validation cohort. **(A, C, E)** Calibration curves for 1-, 3-, and 5-year OS. **(B, D, F)** DCA curves for 1-, 3-, and 5-year OS.

To further assess the robustness and generalizability of the model, we conducted external validation in an independent cohort. The nomogram yielded AUCs of 0.939, 0.778, and 0.865 for predicting 1-, 3-, and 5-year OS, respectively, which were comparable to those observed in the training cohort (**Figure 3E**). The calibration plots and decision curve analyses (**Figures 5A–F**) demonstrated consistent results, further supporting the nomogram’s strong prognostic performance and clinical decision-making utility. Overall, the concordant findings from both internal and external validation confirm that the nomogram offers excellent predictive accuracy and represents a reliable tool for OS risk stratification in CRC patients.

## Discussion

CRC remains a major global health burden, and even after curative resection for stage I–III disease, the risks of recurrence and cancer-related mortality remain substantial (19). In this context, the development of clinically feasible, low-cost, and biologically interpretable prognostic tools is of critical importance. In the present study, we developed and externally validated a novel composite index, the INPR, by integrating PINI and LMR. Our findings demonstrate that INPR provides superior prognostic discrimination compared with either component alone and enables clinically meaningful risk stratification through a user-friendly nomogram, thereby offering a practical framework for individualized postoperative management.

Chronic inflammation has long been recognized as a central hallmark of cancer, playing a pivotal role in colorectal carcinogenesis and progression (20–22). Elevated circulating monocytes serve as precursors of tumor-associated macrophages (TAMs), which accumulate within the tumor microenvironment and are sustained by inflammatory mediators such as IL-6, tumor necrosis factor-α (TNF-α), and prostaglandin E2 (PGE2) (23–27). These cytokines activate persistent NF-κB and STAT3 signaling, establishing a chronic pro-tumorigenic inflammatory state that fosters tumor cell survival, epithelial–mesenchymal transition, and immune evasion (28–30). Conversely, lymphocytes, particularly CD8^+^ cytotoxic T lymphocytes and Th1-polarized CD4^+^ T cells, represent the core of adaptive antitumor immunity. A reduced lymphocyte count is associated with impaired interferon-γ production, diminished granzyme/perforin-mediated cytotoxicity, and enhanced susceptibility to immune checkpoint–mediated exhaustion (31, 32). Consequently, a low LMR biologically reflects a shift in the immune equilibrium toward a monocyte/TAM-dominant, immunosuppressive microenvironment and provides a strong mechanistic basis for its adverse prognostic impact in CRC (33–35).

Therefore, the LMR functions as an integrated marker reflecting the balance between antitumor immune activity and tumor-promoting inflammatory responses. A decreased LMR indicates impaired host immunity accompanied by heightened protumor inflammation, both of which are strongly linked to poor prognosis. Consistent with previous evidence in gastrointestinal cancers, our study demonstrated that a low LMR was significantly associated with shorter OS in patients with CRC (36, 37).

Beyond inflammation, nutritional status represents a critical determinant of host–tumor interactions in colorectal cancer. Serum albumin is not merely an indicator of protein reserves but also a functional antioxidant that buffers reactive oxygen species and modulates systemic redox homeostasis. Hypoalbuminemia is therefore associated with heightened oxidative stress, impaired drug transport capacity, and dysfunctional immune cell signaling. In malnourished states, immune cells undergo profound immunometabolic disturbances, characterized by mitochondrial dysfunction, reduced ATP generation, and impaired amino acid availability, which compromise effector T-cell proliferation and macrophage polarization balance (38–40). Furthermore, cancer-associated metabolic reprogramming, including the Warburg effect and enhanced fatty acid oxidation, creates a nutrient-deprived tumor microenvironment that competitively suppresses antitumor lymphocyte function while favoring the survival and suppressive activity of TAMs (41, 42). This metabolic competition reinforces a vicious cycle of immune suppression and tumor progression. Taken together, these findings provide a robust biological rationale for incorporating nutritional parameters into composite indices such as INPR, which reflect both systemic and local immunometabolic perturbations in CRC (43).

INPR was developed based on the biologically complementary prognostic roles of the PINI and LMR. Although both indices incorporate monocyte counts, they capture distinct and non-redundant biological dimensions of the host–tumor interaction. PINI primarily reflects the interaction between nutritional reserve (serum albumin) and systemic inflammatory burden, thereby representing the host’s metabolic and inflammatory vulnerability. In contrast, LMR reflects the dynamic balance between antitumor adaptive immunity (lymphocytes) and pro-tumor innate inflammatory activity (monocytes), serving as a surrogate indicator of immune equilibrium. We additionally confirmed through formal multicollinearity diagnostics that PINI and LMR did not exhibit significant statistical redundancy, supporting their integration into a composite index. Therefore, INPR is not a mathematically redundant construct, but rather a biologically rational and multidimensional biomarker that captures immune, inflammatory, and nutritional dysregulation in colorectal cancer. This complementary design underlies the superior prognostic performance of INPR compared with single-parameter indices. Biologically, an elevated monocyte burden reflects enhanced recruitment of circulating monocytes into the tumor microenvironment, where they differentiate into TAMs under the influence of cytokines such as IL-6, transforming growth factor-β (TGF-β), and colony-stimulating factor-1 (CSF-1). These TAMs are frequently polarized toward an immunosuppressive M2-like phenotype through activation of canonical signaling pathways, including STAT3, NF-κB, and HIF-1α, thereby promoting angiogenesis, extracellular matrix remodeling, and immune escape via vascular endothelial growth factor (VEGF) and matrix metalloproteinases (44, 45). Simultaneously, lymphopenia reflects impaired cytotoxic immune surveillance, characterized by functional exhaustion of CD8^+^ T cells and natural killer cells through immune checkpoint axes such as PD-1/PD-L1 and CTLA-4, further reinforcing an immunosuppressive tumor niche (46–48). In parallel, hypoalbuminemia represents not only a state of malnutrition but also a surrogate marker of systemic oxidative stress and chronic inflammatory catabolism, which disrupts immune cell metabolism and effector function. Emerging evidence suggests that nutritional deprivation and cancer-associated metabolic reprogramming, including aerobic glycolysis and fatty acid oxidation, competitively impair antitumor immunity, creating a maladaptive feedback loop between immune dysfunction and metabolic stress. Therefore, INPR constitutes a composite biomarker that transcends isolated hematological parameters, reflecting convergent activation of inflammatory signaling, immune exhaustion, TAM-mediated immunosuppression, and metabolic–nutritional dysregulation, which together drive tumor progression and adverse clinical outcomes in CRC.

In recent years, the prognostic landscape of colorectal cancer has increasingly shifted toward multidimensional models that integrate immune contexture, metabolic reprogramming, and TME features. In particular, the concept of “immune-hot” tumors, characterized by abundant cytotoxic lymphocyte infiltration, and “immune-cold” tumors, marked by immune exclusion and macrophage-dominated suppressive niches, has emerged as a powerful framework to explain heterogeneity in clinical outcomes (49–51). Within this paradigm, the balance between lymphocytes and monocytes reflected by LMR may serve as a readily accessible peripheral surrogate of tumor immune phenotypes. Recent studies focusing on hot–cold tumor-related signatures have demonstrated strong prognostic relevance in CRC. Several systemic inflammation- and nutrition-related prognostic scores, including the NLR, PLR, SII, PNI, and controlling nutritional status (CONUT) score, have been proposed for colorectal cancer risk stratification (52–56). However, most of these tools focus on a single biological dimension and therefore fail to comprehensively capture the complex interaction between immune competence, inflammatory burden, and metabolic resilience. In contrast, INPR was specifically designed to integrate complementary immunological and nutritional information into a unified, biologically coherent framework. Our results demonstrated that INPR achieved superior prognostic performance compared with either PINI or LMR alone, supporting the concept that multidimensional indices provide incremental clinical value beyond traditional unidimensional markers.

The principal clinical value of INPR lies in its simplicity, accessibility, and capacity to meaningfully refine postoperative, risk-adapted management in colorectal cancer. Unlike conventional clinicopathological parameters that primarily reflect tumor burden, INPR captures the complex biological interplay between host systemic vulnerability and tumor aggressiveness. In clinical practice, patients classified as high risk (INPR = 0) may benefit from intensified surveillance strategies and consideration of more aggressive adjuvant therapeutic approaches, whereas those in the low-risk category (INPR = 2) may be suitable candidates for de-escalated follow-up and avoidance of overtreatment. In the multivariate model, some traditional clinicopathological variables, such as TNM stage and neural invasion, did not retain independent statistical significance. This finding may be explained by the comprehensive nature of INPR, which integrates systemic nutritional, inflammatory, and immune-related information and may partially capture the downstream biological effects of tumor burden and invasive behavior. Importantly, this does not undermine the clinical relevance of these established prognostic factors but rather highlights the strong integrated prognostic performance of INPR. Although the proportion of patients classified as extremely high risk represented a relatively small subset of the overall cohort, this group consistently exhibited markedly unfavorable survival outcomes in both the derivation and external validation cohorts. This distribution likely reflects the underlying biological heterogeneity of colorectal cancer, rather than statistical instability. Importantly, the reproducibility of this risk pattern across independent cohorts supports the robustness and clinical relevance of INPR in identifying biologically aggressive disease phenotypes. Nevertheless, large-scale, prospective, multicenter studies are still warranted to further confirm the stability, generalizability, and real-world utility of this stratification strategy.

It is also important to consider the potential influence of regional and ethnic characteristics on the observed associations. The present cohort was derived from Xinjiang, a region characterized by unique dietary patterns, cultural habits, and substantial ethnic diversity. Traditional dietary structures, including relatively high intake of animal protein and fat in certain subpopulations, as well as genetic heterogeneity, may influence baseline nutritional indices such as serum albumin and systemic inflammatory status. These population-specific features could affect the distribution of immune–nutritional biomarkers and their prognostic thresholds. Therefore, further validation of the INPR model in geographically and ethnically diverse cohorts is essential to confirm its universal applicability.

Several limitations of the present study should be acknowledged. First, as a retrospective observational study, the possibility of selection bias cannot be completely excluded. Second, this cohort was derived from only two hospitals in Xinjiang, China, which may limit the generalizability of our findings to broader populations; therefore, further validation in larger, multicenter, and multi-ethnic cohorts is warranted. Third, the analysis was restricted to baseline preoperative parameters, and dynamic longitudinal changes in immune–nutritional status were not evaluated. Fourth, detailed information on postoperative adjuvant chemotherapy, including treatment regimens and completion rates, was not uniformly available and therefore could not be incorporated into the multivariable models. Given the established impact of adjuvant therapy on survival in stage II–III colorectal cancer, residual confounding cannot be entirely excluded, and the independent prognostic value of INPR should be interpreted with appropriate caution. Finally, the underlying biological mechanisms through which INPR influences colorectal cancer progression remain incompletely understood and require further mechanistic investigation.

## Conclusion

In summary, our study demonstrated that INPR is a practical and robust prognostic indicator that enables refined risk stratification in patients with stage I–III CRC. Compared with individual markers, INPR exhibited markedly superior predictive performance. When incorporated into a nomogram, INPR provides a promising framework for individualized postoperative management. Future prospective, multicenter investigations are warranted to validate its clinical applicability and to elucidate the biological mechanisms underlying its prognostic value.

## Data Availability

The original contributions presented in the study are included in the article/supplementary material. Further inquiries can be directed to the corresponding author.
